# Generalized Anxiety as a Risk Factor for Dysfunctional Eating Behavior after Obesity Surgery during the COVID-19 Pandemic

**DOI:** 10.3390/ijerph182010890

**Published:** 2021-10-16

**Authors:** Corinna Pfeiffer, Adam Schweda, Lynik Chantal Schüren, Marco Niedergethmann, Jasmin Steinbach, Vanessa Rentrop, Anita Robitzsch, Nora Dörrie, Alexander Bäuerle, Martin Teufel, Eva-Maria Skoda, Benjamin Weismüller

**Affiliations:** 1 Clinic for Psychosomatic Medicine and Psychotherapy, LVR-University Hospital Essen, University of Duisburg-Essen, 45147 Essen, Germany; adam.schweda@lvr.de (A.S.); jasmin.steinbach@lvr.de (J.S.); vanessa.rentrop@lvr.de (V.R.); anita.robitzsch@lvr.de (A.R.); nora.doerrie@lvr.de (N.D.); alexander.baeuerle@uni-due.de (A.B.); martin.teufel@uni-due.de (M.T.); eva-maria.skoda@uni-due.de (E.-M.S.); benjaminmaurice.weismueller@lvr.de (B.W.); 2 Department of Surgery, Alfried-Krupp Hospital Essen, 45131 Essen, Germany; lynik.schueren@krupp-krankenhaus.de (L.C.S.); marco.niedergethmann@krupp-krankenhaus.de (M.N.)

**Keywords:** anxiety, obesity, eating disorder, obesity surgery, COVID-19, mental health

## Abstract

**Purpose:** The present study investigates the impact of obesity surgery on mental health (i.e., eating behavior and distress) during the COVID-19 pandemic. **Methods:** Two hundred fifty-four participants were recruited via social media. One hundred fourteen (44.53%) of them were surgery candidates (waiting for obesity surgery), while 142 (55.46%) had already undergone surgery. Participants who underwent surgery were compared to participants that did not yet undergo surgery in terms of mental burden (depression and anxiety), as well as safety and eating behavior. Further moderation analyses attempted to identify risk factors for increased COVID-19-related dysfunctional eating behavior after surgery. **Results:** Participants who underwent surgery showed generally lower levels of depression and general anxiety on a trend level. Moderation analyses suggested that people with high levels of generalized anxiety actually show more dysfunctional COVID-19-specific eating behavior after obesity surgery. **Conclusion:** On a trend level, obesity surgery appears to attenuate symptoms of generalized anxiety and depression. Yet, surgery patients with high levels of generalized anxiety exhibit even higher levels of dysfunctional eating during the COVID-19 pandemic. It is therefore particularly important to support people at risk.

## 1. Introduction

In March 2020, the World Health Organization declared the spread of the novel coronavirus a worldwide pandemic [1]. Until then, obesity had long been named the worst pandemic of the 21st century and caused more deaths than being underweight worldwide [2]. Since 1975, the prevalence of obesity nearly tripled [2]. Six hundred fifty-nine million adults (18 years and older) were obese in 2017, leading to over 4 million overweight-related deaths, according to the global burden of disease report [3]. Recent studies on COVID-19 showed that obesity worsens the outcome from COVID-19 [4] and that mortality increases as a function of the body mass index (BMI) [5,6,7,8], thus making people suffering from obesity highly at risk for a severe course of disease.

Obese individuals are known to suffer more often than normal-weight controls from a variety of mental comorbidities such as depression, anxiety disorders or eating disorders, and reduced health-related quality of life [9,10,11,12]. A bi-directional link of obesity and depression can be found throughout various studies, showing that obese patients are more depressed and vice versa [13]. Emotional distress and impaired self-management may lead to a loss of structure and a relapse into old behavioral patterns, eventually resulting in weight gain [14]. Additionally, heightened mental stress and problems in emotion regulation trigger impulsive eating symptoms such as binge eating and purging behavior [15,16]. This makes the group of patients suffering from obesity at high risk for elevated levels of psychological burden. Recent studies show the COVID-19 pandemic put a high mental strain not only on the general population but even more so on already psychologically burdened individuals. Patients suffering from obesity seem to be even more at risk for COVID-19-associated psychological burden [17,18]. A retrospective medical chart review showed that since stay-at-home orders were initiated because of the COVID-19 pandemic, patients with obesity reported increased anxiety and depression regardless of infection status [19]. Another study showed that people with obesity had a significant increase in weight, BMI, and changes in the eating psychopathology during the COVID-19 pandemic [20]. These findings not only underlined obese individuals’ risk of various somatic and psychological comorbidities, but also suggested a high-risk status in the current COVID-19-pandemic.

In Western countries, obesity surgery is the most common treatment for patients with BMI ≥ 40 kg/m^2^ or BMI ≥ 35 kg/m^2^ who also suffer from obesity-related comorbidities and did not respond to behavioral treatment, exercises, and nutritional treatment [21,22]. A recent RCT found that obesity surgery candidates seem to suffer from equally elevated levels of depression as psychotherapy inpatients, making this group also prone to heightened psychological strain during the current pandemic [23]. Findings from before the COVID-19 pandemic showed that for most of these patients, mental health improves after obesity surgery even if the mechanism and the psychological factors remain unclear [24,25]. Although dysfunctional eating behaviors decreased directly after obesity surgery between the first and third year after the intervention, dysfunctional eating behavior significantly increases again [26]. Weight loss as a result of obesity surgery does not mean an improvement in mental health at the same time, as the expectations of a life-changing measure can be exaggerated and frustrating [27].

Literature is lacking on the impact of obesity surgery in obese individuals concerning eating behavior and the psychological distress during the COVID-19 pandemic. Restrictions in social life due to quarantine measures, physical distancing, and COVID-19-related fear may pose a special burden for this vulnerable patient group. The aim of the current study was to investigate to what extent obesity surgery affects COVID-19-related eating behavior, generalized anxiety, depression, and psychological distress. It is hypothesized that obesity surgery significantly affects dysfunctional eating behavior, bulimic eating behavior, anxiety, and depression during the current COVID-19 pandemic. More precisely, patients probably suffer less from dysfunctional COVID-19-specific eating behaviors, anxiety, and depression after they obtained an obesity surgery compared to a group of obese people that are still awaiting such a surgical measure.

## 2. Method

### 2.1. Participants and Procedure

Participants were recruited online from a German obesity center of excellence and via social media from 10 May to 7 July 2020. Two hundred fifty-four participants (223 female, 31 male) completed the study: 114 participants (99 female, 15 male) did not (yet) have an obesity surgery, while 140 (124 female, 16 male) did already undergo obesity surgery. Mann–Whitney tests did not reveal significant gender differences between the with and without surgery groups (U = 8070.00, *p* = 0.944), but difference in age between surgery groups was significant (U = 6764.50, *p* = 0.019). Table 1 lists all sociodemographic and medical data, including age and gender distributions of both groups. Electronic informed consent was given and confirmed by all participants. Participation was voluntary and anonymous, and participants could withdraw from the study at any time. The proposed study was conducted in accordance with the Declaration of Helsinki, and the local Ethics Committee of the Medical Faculty approved this study (20-9307-BO).

### 2.2. Measures

Demographic information such as the participant’s age (see above), gender (male; female; other), community size, education, and their current occupation were assessed. Then, validated instruments and self-generated scales assessed psychological states and psychological reactions to COVID-19. Weight and height were also assessed. Mental burdens during the previous two weeks were measured using the Patient Health Questionnaire-2 (PHQ-8, measuring depression symptoms with two items on a four-point Likert Scale [28,29]) and the Generalized Anxiety Disorder-7 (GAD-7, measuring generalized anxiety using seven items on a four-point Likert Scale [30,31]). To measure specific COVID-19-related fear, one single seven-point Likert-scaled item was used (for further information see [32]). Additionally, participants were asked about changes in their general eating behavior since the start of the COVID-19 pandemic in Europe. In 10 self-generated items, participants indicated whether they observed themselves eating more or less, shopping for more groceries, eating more fast food, and eating larger portions on a seven-point Likert Scale (see Supplementary Material for specific wording and factorial analyses). These items were then summarized in one scale indicating dysfunctional COVID-19-specific eating behavior (DCSEB).

### 2.3. Data Analysis

To assess normality, distributions of all analyzed variables were visually assessed and tested using Kolmogorov–Smirnov tests. Indeed, this approach revealed that all of the tested variables significantly deviated from the normal distribution in both sample groups (all *p*s < 0.007). Accordingly, predominantly non-parametric as well as robust approaches were applied throughout the entire analysis. In order to extract a meaningful scale to express a rise in increased and more unhealthy food intake during the COVID-19 pandemic, a factorial analysis was applied to the 10 items measuring COVID-19-specific eating behavior (DCSEB). Self-generated items for *dysfunctional safety behavior* have been intensively discussed in previous studies by our group (please see [33]). Cronbach’s α for *dysfunctional safety behavior* in the current sample was 0.794.

To test univariate associations between COVID-19-related variables—*generalized anxiety*, *depression*, *dysfunctional COVID-19-related eating behavior*, and *dysfunctional safety behavior*—Spearman correlation coefficients were computed. To further explore whether obesity surgery had an influence on the respective psychopathological dimension (PHQ-8, GAD-7), COVID-19-related fear, and dysfunctional COVID-19-related eating behavior (DCSEB), group differences (with vs. without surgery) were assessed via Mann–Whitney U tests. Separate robust regression analyses—as implemented in the R package *robustbase* [34]—were then computed to assess whether the associations between *DCSEB* and COVID-19-related fear, depression, and anxiety symptoms (PHQ-8 and GAD-7) are moderated by obesity surgery. To do so, the respective psychological variable, the group variable (*with* and *without* obesity surgery), as well as their interaction coefficients were regressed on *DCSEB*. A full summary of regression coefficients is provided in the Supplemental Materials. The data were analyzed using IBM Statistics SPSS 26 (New York, NY, USA) and R (3.6.3).

## 3. Results

First, a factorial analysis was performed to extract an interpretable measure of increased and more unhealthy food intake during the COVID-19 pandemic (“dysfunctional COVID-19-specific eating behavior”, DCSEB). A parallel analysis, as well as Velicer’s minimum average partial (Velicer, 1976), were applied to extract the optimal number of factors. Both analyses convergingly indicated the existence of one factor. Within this one factor (proportion of explained variance = 36%), four items reached standardized factor loadings of above 0.6 (Awang, 2014, Hair, 2008; see Supplemental Material). These items assess whether the individual started to eat larger portions more frequently in an unhealthier fashion, and whether they fell back into old eating patterns. Kaiser–Meyer–Olkin measures of sampling adequacy indicate values of above 0.8 for each item; sum scores were applied to subsequently summarize the scale.

Spearman correlation analyses revealed significant associations between *DCSEB* and *COVID-19-related fear* (*r* = 0.167; *p* = 0.008), *DCSEB* and *generalized anxiety* (*r* = 0.396; *p* < 0.001), and *DCSEB* and depression symptoms (*r* = 0.496; *p* < 0.001). For an overview of all correlation coefficients, see Tables S1 and S2 in the supplementary online material. To explore possible effects of obesity surgery on the psychopathological states and eating behavior, Mann–Whitney U tests were computed to identify differences between groups (*with* and *without* surgery) in each of the psychometric scales mentioned above. These Mann–Whitney U tests revealed no significant differences in the tested variables: *COVID-19-related fear* (*W* = 8288, *p* = 0.739), *dysfunctional safety behavior* (*W =* 8695.5, *p* = 0.305), and *DCSEB* (*W* = 8431.5, *p* = 0.566). However, *p*-values approached significance at α = 0.05 for the comparisons between participants with and without obesity surgery in *generalized anxiety* (*W* = 9180, *p* = 0.064) *and depression symptoms* (*W* = 9186, *p* = 0.057), and participants who underwent obesity surgery exhibited lower levels in each of these dimensions. Table 2 lists the psychometric data for the obesity patients *with* and *without* obesity-specific surgery.

To assess whether obesity surgery moderates the relationship between the above-described psychological dimensions and *DSCEB*, robust regression analyses were conducted for each possible predictor, using *group* (*with* vs. *without* surgery) as a moderator and *DCSEB* as the dependent variable. The strongest interest was to reveal unconditional relationships so that one regression model was computed for each predictor.

**Figure 1:**
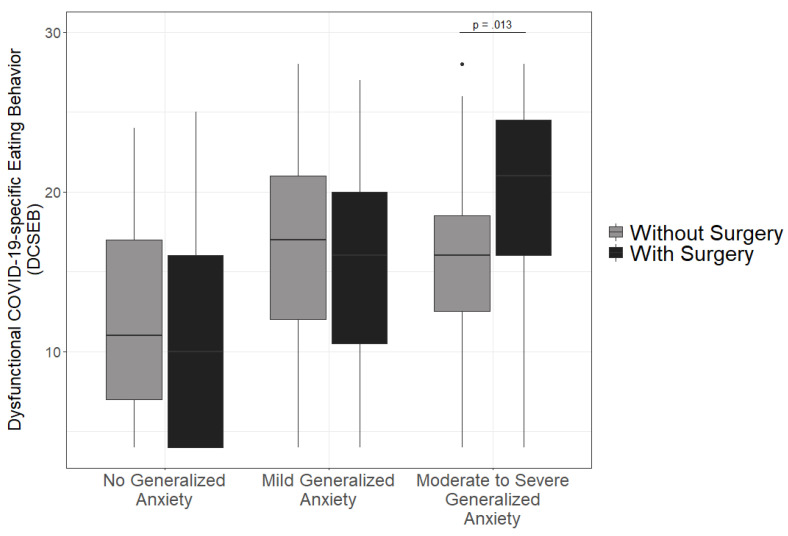
Generalized anxiety as a risk factor for increased levels of dysfunctional COVID-19-specific eating after obesity surgery. Group-wise box-plots indicate medians and interquartile ranges (see supplemental material for illustration of non-splitted continuous data). While for participants with no or mild manifestations of generalized anxiety (GAD-17 scores of 0 to 4, or 5 to 9, respectively), no increase in DCSEB is notable, and individuals with moderate to severe levels of anxiety (GAD-7 > 9) show increased DCSEB after obesity surgery. Whiskers extend to the most extreme data point unless there are data 1.5 inter-quartile-ranges away from the first or the third quartile, respectively. Data points beyond that are shown as a black dot.

This moderator analysis revealed a significant interaction between the predictors *generalized anxiety* and group (with vs. without surgery, *b* = 0.289; *p* = 0.028, see supplemental material for illustration of the marginal effects) on *DCSEB*. The regression coefficient for generalized anxiety turned out significant (*b* = 0.227, *p* = 0.025). No differences occurred in the direct comparison between patients with and without surgery (*b* = −0.003, *p* = 0.983). The regression model accounted for 16.6% of variance. This pattern—and particularly the interaction between group and generalized anxiety—remained robust after conditioning on age, gender, and education. No other significant interaction appeared in these regression models (see supplemental online material). To further illustrate this effect, participants were divided according to common cutoffs for the GAD-7, namely participants who show no anxiety (GAD-7 score below five), people who exhibit mild anxiety (GAD-7 scores from five to nine), and participants who report moderate to severe anxiety (GAD-7 scores from 10 to 21, see [35]). The moderating effect of generalized anxiety on DCSEB before and after surgery is shown in Figure 1. Corroboratory results from a further robust regression analysis that included the categorized GAD-7 values (no anxiety vs. mild anxiety vs. moderate to severe anxiety), the group variable (with vs. without surgery), and their interaction term also indicated that while levels of DCSEB remained unchanged for individuals with surgery compared to individuals without surgery in participants with low and mild anxiety levels, participants with high anxiety showed even more DCSEB after surgery (interaction term between surgery [reference: without surgery] and GAD-7 [dummy: mild anxiety with reference: no anxiety]: *b* = 0.049, *se* = 0.286, *t* (250) = 0.170, *p* = 0.865; interaction term between surgery [reference: without surgery] and GAD-7 [dummy: moderate and severe anxiety with reference: no anxiety]).

## 4. Discussion

The present study is, to our knowledge, one of the first to investigate the influence of obesity surgery on psychological burden in patients with obesity. We analyzed possible effects of obesity surgery during the COVID-19 pandemic on mental health burden (PHQ-8, GAD-7, COVID-19-related fear, DCSEB) by comparing patients *with* and *without* obesity surgery. In general, group comparisons showed no differences between these groups, suggesting that the surgery did not affect any psychological state. The two groups only differ at a trend level in generalized anxiety and depressive symptoms, suggesting a slightly increased burden in individuals without surgery. More precisely, in individuals that do not suffer (much) from generalized anxiety, DCSEB does not differ across obesity surgery groups (*with* or *without*). In contrast, people that do suffer from generalized anxiety differ in their DCSEB depending on their obesity surgery status (*with* or *without*), with more DCSEB in people with a surgery. Accordingly, generalized anxiety moderates DCSEB after obesity surgery. The interaction between generalized anxiety and history of obesity surgery shows that people with obesity already suffering from generalized anxiety symptoms and/or bulimic eating seem to suffer even more compared to people who already underwent the surgery during the pandemic. Thus, generalized anxiety seems to be a risk factor for dysfunctional eating behavior after obesity surgery during the COVID-19 pandemic.

The COVID-19 pandemic still has a deep impact on our social life, quality of life, and mental health [18,36]. COVID-19-related fear and generalized anxiety, particularly for vulnerable individuals, play decisive roles in mental health during the pandemic [17,32,37,38].

Meanwhile, anxiety is linked to all types of eating disorders [39,40] and is the most prevalent emotion obese people with a binge eating disorder experience prior to a binge [41]. The frequency of binge eating episodes is higher in patients with higher anxiety scores than in grade III obesity patients [42,43,44]. Thus, negative emotions seem to be controlled and regulated by activating the neuronal reward system during the consumption of palatable foods [45,46].

In times of increased mental distress caused by the COVID-19 pandemic, the access to protective resources could be difficult so that people may fall back into old behaviors using the same emotion regulation strategies as before the pandemic. This means that, on one hand, obesity surgery does not offer an increased stress resilience during the COVID-19 pandemic and, on the other hand, mentally stable people who underwent obesity surgery will continue to do so even in times of crisis. For those who already suffer from mental illnesses or instability, mental decompensation can occur more quickly in times of mental distress because of the COVID-19 pandemic. Thus, psychosocial evaluation and support is of particular importance for obesity patients prior to surgery in order to avoid possible dysfunctional stress regulation, consecutive weight gain, and eventually the deterioration of long-term results [47,48]. Before the pandemic, studies showed that in most patients, mental health improved after obesity surgery even in patients with previous psychiatric illnesses. However, underlying mechanisms and psychological factors remain unclear [24,25,49]. Individual psychological resources seem to be one important protective factor for mental health in people suffering from obesity [50].

These results once again underline the need and importance for structured interdisciplinary aftercare in the group of obesity surgical patients suffering from psychological distress during the COVID-19 pandemic, including psychotherapeutic and psychosocial support. Low-threshold support services are required, such as evidence-based cognitive behavioral emotion regulation skills like stress management, meditation, physical exercise, stimulus control, etc. These could increase the likelihood that mental illnesses will turn chronic [51]. Emerging E-mental health interventions could be a helpful tool and an addition to support people with psychological burden [52]. Special consideration should be given to find tailor-made interventions and aftercare support towards patients who continue to show compensatory eating behavior postoperatively in the context of psychological distress.

### 4.1. Limitations

First, this study was a cross-sectional study, not a repeated-measurements design, so no causality can be directly inferred from the data regarding obesity surgery. However, as many other relevant variables have been measured and controlled across both groups, moderation effects of the surgery in the present sample can still be interpreted. Then, the presented data were collected by an online questionnaire, which necessarily holds some limitations. For instance, participant response rates cannot be controlled so that a participant bias seems plausible. In consequence, this lack of participant control may influence the results’ generalizability. Furthermore, the possibility of selection bias should be considered.

Last, psychological COVID-19-specific traits reported here were not measured by validated instruments, simply because none existed to that date. Ahorsu et al. [53] created the first questionnaire to assess COVID-19-related fear after the present survey had been launched—the Preventive COVID-19 Behavior Scale (PCV-19BS, see [53,54]). Thus, COVID-19-related fear and DSCED were self-generated items or at least adapted to assess COVID-19-specific traits. As can be seen in previous studies [17,18,33,54], however, this COVID-19-related fear item qualifies relatively well to assess fear, but not generalized anxiety, at the time of the pandemic. Despite being the first study on the influence of obesity surgery on COVID-19 distress, the study is limited in terms of gender differences. Of course, additional factors such as the connection to an obesity center should be considered.

### 4.2. Conclusions

After obesity surgery, patients can be at risk to be additionally challenged by the pandemic. Psychosocial support is of particular importance for people who already suffer from mental illness to achieve stress resistance, mental health, and weight goals and not to relapse in overcome behaviors. Therefore, it is important to ensure medical, psychological, and surgical care and support for patients with obesity during the COVID-19 pandemic to assure equal opportunities regarding upcoming health challenges.

## Figures and Tables

**Table 1:** Sociodemographic characteristics separately for both groups (with and without surgery).

	Without Surgery		With Surgery		
	N	%	N	%	*p*-Value
**Sex**					
Female	99	86.8	124	87.3	
Male	15	13.2	16	11.3	0.821
**Age**					
18–24 years	5	4.4	1	0.7	
25–34 years	26	22.8	27	19	
35–44 years	45	39.5	52	36.6	
45–54 years	26	22.8	30	21.1	
55–64 years	11	9.6	25	17.6	
65–74 years	1	0.9	5	3.5	
≥75 years	0	0	2	1.4	0.100
**Marital status**					
Single	24	21.1	30	21.1	
Married	61	53.5	70	49.3	
In a relationship	16	14	27	19	
Divorced/separated	12	10.5	8	5.6	
Widowed	1	0.9	4	2.8	0.371
**Educational level**					
University education	12	10.5	20	14.1	
Higher education entrance qualification	33	28.9	32	22.5	
Higher secondary education	42	36.8	63	44.4	
Lower secondary education	22	19.3	25	17.6	0.460
**Employment**					
Employed	63	55.3	98	69	
Not employed	37	32.5	44	31	0.402
**City size (Population)**					
100,000 residents	65	57	85	59.9	
20,000 residents	21	18.4	25	17.6	
5000 residents	18	15.8	14	9.9	
<5000 residents	10	8.8	18	12.7	0.428
**Mental illness**					
yes	34	29.8	42	29.6	
no	80	70.2	100	70.4	
					1.000
**Somatic illness**					
none	15	13.2	29	20.4	0.172
Cardiovascular disease	11	9.6	6	4.2	0.271
Diabetes mellitus	23	20.2	28	19.7	1.000
Chronic respiratory disease	24	21.1	26	18.3	0.695
Hypertension	56	49.1	47	33.1	0.014
Intermittent claudication	1	0.9	3	2.1	0.776
Sleep apnea	21	18.4	25	17.6	0.996
Lip-metabolic disorder	12	10.5	14	9.9	1.000
Articular gout	11	9.6	13	3.2	1.000
Hypothyroidism	35	30.7	44	31	0.142
Polycystic ovary syndrome	8	7	11	7.7	1.000
Arthropathy	41	36	49	34.5	0.912
other	18	15.8	26	18.3	0.715
**Total**	114	100	142	100	

**Table 2:** Psychometric data for the obesity patients with and without an obesity-specific surgery. Mean sum scores and standard deviations (in parentheses) are listed.

	Without Surgery	With Surgery
N	114	142
Weight	132.72 (31.57)	101.43 (22.26)
Body Mass	45.59 (10.49)	35.49 (8.96)
COVID-19-related fear	4.21 (1.95)	4.14 (1.89)
Generalized anxiety (GAD-7)	7.21 (5.19)	6.37 (6.00)
Depression symptoms (PHQ-8)	9.02 (5.19)	8.00 (6.58)
Dysfunctional safety behavior	3.27 (1.57)	3.11 (1.61)
Dysfunctional COVID-19-specific eating behavior (DCSEB)	14.69 (6.23)	14.22 (7.50)

Note: Generalized anxiety was measured by GAD-7 (7 items, 4-point Likert scale, cut-off mild = 5, cut-off moderate = 10); depression symptoms were measured by PHQ-8 (8 items, 4-point Likert scale, cut-off ≥ 10), COVID-19-related fear, dysfunctional safety behavior, Dysfunctional COVID-19-specific eating behavior (DCSEB, see Supplementary online Material). Body mass was computed using the formula weight in kg/(height in m)^2^.

## Data Availability

The raw data will be made available upon reasonable request to the correspondent author.

## Supplementary Materials

The following are available online at www.mdpi.com/article/10.3390/ijerph182010890/s1, Table S1: The full reports of our regression analyses, Table S2: Correlation Matrix.
