# Supplementary material for: Generalized Anxiety as a Risk Factor for Dysfunctional Eating Behavior after Obesity Surgery during the COVID-19 Pandemic
Source: Int J Environ Res Public Health. 2021 Oct 16;18(20):10890. doi: 10.3390/ijerph182010890 (PMC8536045; doi:10.3390/ijerph182010890)
Supplement: Supplementary file 1 [file ijerph-18-10890-s001.zip › ijerph-1406020-supplementary.pdf]

Supplemental Material

Generalized anxiety as a risk factor for dysfunctional eating behavior after obesity surgery during the COVID-19 pandemic

Pfeiffer et al. (2021)

Supplemental methods and results

In order to offer further information on our data, we provide the reader with additional material concerning the items used as well as the data analysis. Table 1a-c shows the full reports of our regression analyses. Here, the continuous variables PHQ-2, GAD-7, and COVID-19 related fear, as well as the dummy variable „obesity surgery“ (without vs. with surgery, reference category: without surgery) plus their interaction term were used as regressors. The dependent variable was always the DCSEB. Table 2 shows the bivariate Spearman correlation coefficients. Finally, the last section lists the items we used for construction of the DCSEB, the dysfunctional safety behavior scales, as well as the item to measure COVID-19 related fear.

Table S1a

Predictors	*Estimates*	*CI*	*p*
(Intercept)	0.01	-0.15 – 0.17	0.907
Depression (PHQ-8)	0.42	0.23 – 0.61	**<0.001**
Surgery (ref: Before)	0.02	-0.21 – 0.25	0.878
Interaction Term	0.17	-0.08 – 0.41	0.182
Observations	256		
R^2 / R^2 adjusted	0.257 / 0.248		

Table S1b

Predictors	*Estimates*	*CI*	*p*
(Intercept)	0.02	-0.15 – 0.19	0.846
Gen. Anxiety (GAD-7)	0.23	0.03 – 0.42	**0.025**
Surgery (ref: Before)	-0.00	-0.24 – 0.24	0.982
Interaction Term	0.29	0.03 – 0.55	**0.028**
Observations	256		
R^2 / R^2 adjusted	0.166 / 0.156		

Table S1c

Predictors	*Estimates*	*CI*	*p*
(Intercept)	0.03	-0.14 – 0.20	0.727
Fear of COVID-19	0.15	-0.03 – 0.33	0.109
Surgery (ref: Before)	-0.06	-0.32 – 0.20	0.657
Interaction Term	0.06	-0.21 – 0.34	0.648
Observations	256		
R^2 / R^2 adjusted	0.031 / 0.020		

Table S2: Correlation Matrix

	Depression (PHQ-8)	Generalized Anxiety (GAD-7)	DCSEB
Depression (PHQ-8)			
Generalized Anxiety (GAD-7)	0.86****		
DCSEB	0.50****	0.40****	
COVID-19-related fear	0.21***	0.21***	0.17**

Scale *DCSEB (Dysfunctional COVID-19-specific Eating Behavior,* bold text represents items that were included in the DCSEB scale*)*

Since the outbreak of the COVID-19 pandemic…

1. **… I've been eating more often than before.**
2. **… I've been eating larger meals than before.**
3. **… I've been eating more unhealthy food than before.**
4. … my shopping behavior has changed due to hygiene measures in grocery stores.
5. … I buy more groceries than before.
6. … I prepare more food using basic ingredients.
7. … I eat less fast food.
8. … I take additional vitamin supplements.
9. **… I have fallen back into „old behaviors".**
10. … my food intake has become more important to me.

Scale *Dysfunctional Safety Behavior*

1. I have bought larger quantities of basic food (flour, sugar, noodles, rice, and canned food) or will buy more in the near future.
2. I have bought larger quantities of hand disinfection/soap/similar or will buy more in the near future.
3. I have bought larger quantities of toilet/hygiene articles or will buy more in the near future
4. I have become more selfish in my behavior.

COVID-19-related fear

- I worry about COVID-19.
